# Trends of Publications on Single- and Double-Bundle ACL Reconstructions over the Last 20 Years: A Bibliometric Analysis of the PubMed Literature

**DOI:** 10.1007/s43465-023-00977-5

**Published:** 2023-08-29

**Authors:** Riccardo D’Ambrosi, Srinivas B. S. Kambhampati, Karthik Vishwanathan, Abhishek Vaish, Raju Vaishya

**Affiliations:** 1 IRCCS Ospedale Galeazzi – Sant’Ambrogio, Milan, Italy; 2 Dipartimento di Scienze Biomediche per la Salute, Università degli Studi di Milano, Milan, Italy; 3 Sri Dhaatri Orthopaedic, Maternity and Gynaecology Center, SKDGOC, Vijayawada, Andhra Pradesh India; 4 Parul Institute of Medical Sciences and Research, Parul University, Limda, Waghodia, Vadodara, Gujarat India; 5 Indraprastha Apollo Hospitals, New Delhi, India

**Keywords:** Anterior cruciate ligament, ACL, Bibliometry, Single bundle, Double bundle, Knee

## Abstract

**Purpose:**

To improve the clinical outcomes of anterior cruciate ligament reconstruction (ACLR), there have been attempts to reproduce anatomic reconstruction by modifying the single-bundle (SB) and double-bundle (DB) techniques. Although DB ACLR restores better rotational control compared to SB ACLR, it is still debatable whether there are higher clinical outcomes in favor of DB ACLR. We aimed to study the trends of publications on SB and DB ACLR techniques over the last 20 years.

**Methods:**

For this bibliometric study, we performed a PubMed search on 31/05/2022 with a well-defined search strategy. The articles were downloaded into Excel software, and citations were determined from the iCite website for PubMed. The analysis was performed using SPSS software version 28.0.1. Data mining was performed using Orange software, Mac version 3.32.0, from the titles of all articles and each group of SB and DB ACLR. The output is presented as word clouds.

**Results:**

A total of 10,530 publications were identified, of which 9699 publications (92.1%) pertained to SB-ACLR and 831 publications (7.9%) to DB-ACLR. There was a steady increase in the publications on SB-ACLR until 2012, followed by a steep increase that peaked in 2021. The highest number of publications on DB-ACLR was in 2012 (*n* = 76; 9.1%). The mean citations per year for SB-ACLR and DB-ACLR were 2.87 ± 4.31 and 2.74 ± 3.17, respectively. The most prolific journals publishing on this topic were Knee Surgery Sports Traumatology Arthroscopy, American Journal of Sports Medicine, and Arthroscopy. The top three articles that received the maximum number of citations were from Japanese authors.

**Conclusion:**

The number of publications related to SB-ACLR was significantly higher than that related to DB-ACLR in the last 20 years. The publications related to DB-ACLR have decreased in the recent past, after reaching a peak in 2012. The citations per year of SB-ACLR and DB-ACLR were similar.

## Introduction

The primary objective of anterior cruciate ligament reconstruction (ACLR) is to effectively restore knee stability and facilitate the resumption of sports activities and everyday functional activities among patients [1–3]. Nevertheless, a number of studies have indicated that ACLR does not fully restore the typical structure and functionality of the knee [4–8]. Less than 70% of patients return to their preinjury sports activity, and less than 60% of competitive athletes return to sports [9–11]. Several issues remain regarding the development of posttraumatic knee osteoarthritis (OA) after ACLR. The literature reports that the prevalence of radiographic OA ranges from 40 to 90% at 7–12 years after surgery [12–15].

Throughout the years, numerous surgical procedures have been reported with the aim of optimizing the anatomical positioning of ACLR. These techniques involve the creation of tunnels inside the anatomical footprints of the tibial and femoral insertions of the natural ligament, ultimately seeking to optimize surgical outcomes [16–18].

The anatomic surgical strategies for ACLR include single-bundle (SB), in which a single graft is used to replace both bundles of the ACL, or double-bundle (DB) ACLR, in which separate grafts are used to replace both bundles (posteromedial and anterolateral) of the ACL. Biomechanical studies have found that DB ACLR restores better rotational control than SB ACLR [19, 20]. However, the current literature, in particular, meta-analyses of levels 1 and 2, report inconsistent conclusions [19, 21]. Some studies concluded that SB ACLR results in lower rotational and anterior laxity [22, 23]. However, only a few have demonstrated higher clinical outcomes in favor of DB ACLR [24, 25].

According to a recent systematic review, it was observed that American orthopaedic surgeons currently favor the technique of independent tunnel drilling in single-bundle anterior cruciate ligament reconstruction (SB ACLR) [26]. The decisions made in this context are grounded on evidence-based practises, relying on the outcomes derived from randomized controlled trials. These trials provide support for the recommendations of the American Academy of Orthopaedic Surgeons (AAOS), which are considered to be of the highest level.

Bibliometric analysis is a quantitative approach that employs mathematical and statistical methods to examine the bibliographic characteristics of scholarly literature within a specific academic domain. Various analytic tools are available for conducting research, such as CiteSpace, Pajek, UCINET, and VOSviewer. Among these tools, CiteSpace has gained significant popularity [27].

These software applications have been extensively utilized across various study disciplines to analyze knowledge structures, transition patterns, and emerging trends. A number of bibliometric studies have lately been published in journals with high impact factors. Nevertheless, there is a scarcity of bibliometric articles within the ACLR study domain [28].

The purpose of this study is to analyze and compare the trends of publications on SB and DB ACLR techniques over the last 20 years. We hypothesized that publications on DB ACLR have been decreasing in the recent past.

## Methods

### Search Strategy

We performed a PubMed search on 31/05/2022 with the following search strategy:

(("anterior cruciate ligament"[MeSH] OR "anterior cruciate ligament injuries"[MeSH] OR ACL) AND (Knee)) AND (reconstruction) with filters: from 2002 to 2021. We retrieved 10,525 articles. These included SB and DB publications. A search with the above strategy and including “AND Double bundle” retrieved 831 articles. SB publications were obtained by including “NOT double bundle” in the strategy. These were considered single-bundle ACLR articles, and there were 9699 articles with this strategy.

### Data Collection

The following information was extracted from the included studies: type of ACLR (SB or DB), year of publication, name of the journal, PMID numbers of the articles, first author, name of the Institution and the Country where the work was conducted. The articles were downloaded into Excel software (MS Office 365), and citations were determined from the iCite website (https://icite.od.nih.gov/) for PubMed. Analysis was performed using SPSS software version 25.0.1. Data mining was performed using Orange software, Mac version 3.32.0 (https://orangedatamining.com/) from the titles of all articles and each group of SB and DB ACLR. The output is presented as word clouds.

### Relative Citation Ratio (RCR)

The RCR is a newly developed metric that was introduced to prevent disparity between young researchers in comparison to senior researchers and also to correctly gauge the influence of the scientific work by comparing it with other published work in the same field of work [29, 30]. The RCR value of 1 is considered to be the recommended standard as it is equivalent to NIH-funded scientific publications in the same field of work [31].

### Statistical Methods

The parameters evaluated were the total number of citations (citation count), Relative Citation Ratio (RCR), citations per year, expected citations per year, field citation rate and NIH percentile.

The pattern of data distribution was analyzed using the Kolmogorov–Smirnov test and accordingly, parametric (independent samples *t* test) or non-parametric test (Mann–Whitney *U* test) was used for statistical analysis. Independent samples *t* test was used to evaluate the difference of RCR in SB-ACLR and DB-ACLR and Mann–Whitney *U* test was used to evaluate the differences in citation count, citations per year, expected citations per year, field citation rate and NIH percentile. We performed a year-wise comparison of the total number of publications of SB-ACLR and DB-ACLR to ascertain the trend in publications. We sought to determine the ten most influential articles worldwide on SB-ACLR and DB-ACLR. This was determined using the total citation count of the article.

## Results

### Literature Search

A total of 10,530 publications were identified, out of which 9699 publications (92.1%) pertained to SB-ACLR and 831 publications (7.9%) pertained to DB-ACLR. Checking for duplicates was performed using PMID numbers, and there were no duplicates.

### Number of Publications and Trend

**Fig. 1:**
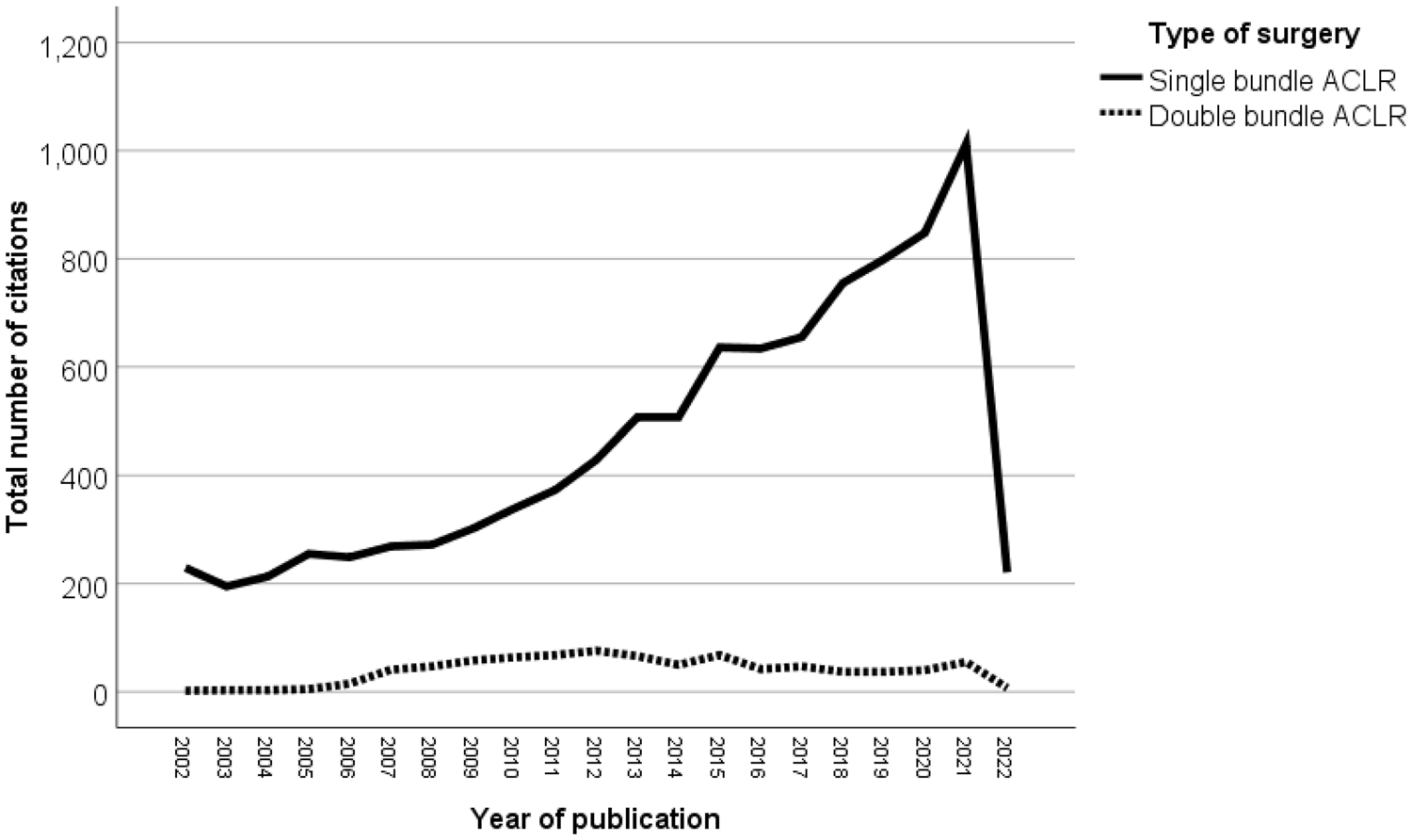
A comparative trend of publications on single-bundle ACL reconstruction (SB-ACLR) and double-bundle ACL reconstruction (DB-ACLR)

There were more publications on SB-ACLR than on DB-ACLR (Fig. 1 and Table 1). There was a steady increase in the number of publications of SB-ACLR until 2012, followed by a steep curve that peaked in 2021. The highest number of publications on DB-ACLR was in 2012 (*n* = 76; 9.1%), followed by 2011 (*n* = 68; 8.2%) and 2015 (*n* = 68; 8.2%). The highest peak in 2012 for DB-ACLR was preceded by a gradual increase in the number of publications and followed by a gradual decline in the number of publications.

**Table 1:** Comparison of the numbers of publications on single-bundle ACL reconstruction (SB-ACLR) and double-bundle ACL reconstruction (DB-ACLR)

Year of publication	SB-ACLR % calculated based on 9694 publications of SB-ACLR *N* = 9699	DB-ACLR % calculated based on 831 publications of DB-ACLR *N* = 831
2002	229 (2.4%)	2 (0.2%)
2003	195 (2%)	3 (0.4%)
2004	213 (2.2%)	3 (0.4%)
2005	255 (2.6%)	5 (0.6%)
2006	249 (2.6%)	15 (1.8%)
2007	269 (2.8%)	41 (4.9%)
2008	272 (2.8%)	47 (5.7%)
2009	302 (3.1%)	58 (7.0%)
2010	339 (3.5%)	64 (7.7%)
2011	373 (3.8%)	68 (8.2%)
2012	429 (4.4%)	76 (9.1%)
2013	507 (5.2%)	66 (7.9%)
2014	507 (5.2%)	50 (6%)
2015	636 (6.6%)	68 (8.2%)
2016	634 (6.5%)	42 (5.1%)
2017	655 (6.8%)	47 (5.7%)
2018	755 (7.8%)	37 (4.5%)
2019	799 (8.2%)	37 (4.5%)
2020	848 (8.7%)	40 (4.8%)
2021	1012 (10.4%)	55 (6.6%)
2022—up to 22/6/2022	221 (2.3%)	7 (0.8%)

### Journals with Most Articles

The journal with the highest number of publications on SB-ACLR was Knee Surgery Sports Traumatology Arthroscopy (*n* = 1398; 14.4%), followed by American Journal of Sports Medicine (*n* = 993; 10.2%) and Arthroscopy: The Journal of Arthroscopic and Related Surgery (*n* = 819; 8.4%). The journal with the highest number of publications on DB-ACLR was Knee Surgery Sports Traumatology Arthroscopy (*n* = 194; 23.3%), followed by Arthroscopy: The Journal of Arthroscopic and Related Surgery (*n* = 137; 16.5%) and American Journal of Sports Medicine (*n* = 105; 12.6%).

### Most Cited Articles

For SB-ACLR, the mean number of citations was 22.2 ± 44.22 (0–1346), and the median number of citations was 9 (interquartile range: 2, 25). A total of 370 out of 9699 publications (3.8%) on SB-ACLR were classic papers with more than 100 citations. The highest number of citations (*n* = 1346) was that of the review article published in the American Journal of Sports Medicine (PMID: 17761605). The second highest number of citations (*n* = 1119) was the validation study on the Knee Injury and Osteoarthritis Outcome Score n published in Health and Qualify of Life Outcomes (PMID: 14613558). The third highest number of citations (*n* = 893) was for the cohort study published in Arthritis and Rheumatism (PMID: 15476248). All the top three cited papers were from Lund University, Sweden. The bibliometric details of the top ten publications on SB-ACLR are presented in Table 2.

**Table 2:** The 10 most popular articles on single-bundle ACL reconstruction (SB-ACLR)

Number	Authors, Journal, year	PMID	Country	Institution	Citation count
1	Lohmander et al., Am J Sports Med, 2007	17761605	Sweden	Lund University, Lund	1346
2	Roos et al., Health Qual Life Outcomes, 2003	14613558	Sweden	Lund University, Lund	1133
3	Lohmander et al., Arthritis Rheum, 2004	15476248	Sweden	Lund University, Lund	896
4	Paterno et al., Am J Sports Med, 2010	20702858	USA	Cincinnati Children's Hospital Medical Center, Cincinnati, Ohio	682
5	Ardern et al., Br J Sports Med, 2011	21398310	Australia	La Trobe University, Bundoora, Victoria	615
6	Yagi et al., Am J Sports Med, 2002	12238998	USA	University of Pittsburgh, Pittsburgh, Pennsylvania	591
7	Ardern et al., Br J Sports Med, 2014	25157180	Australia	La Trobe University, Bundoora, Victoria	545
8	Øiestad et al., Am J Sports Med, 2009	19567666	Norway	Ullevaal University Hospital, Oslo	508
9	Mall et al., Am J Sports Med, 2014	25086064	USA	USA Cartilage Restoration Center of St Louis, St Louis, Missouri + Rush University Medical Center, Chicago, Illinois	481
10	Grindem et al., Br J Sports Med, 2016	27162233	Norway USA	Norwegian School of Sport Sciences, Oslo + Oslo University Hospital, Oslo + University of Delaware, Newark, Delaware	471

For DB-ACLR, the mean number of citations was 26.6 ± 40.16 (0–352), and the median number of citations was 12 (interquartile range: 4, 33). Thirty-seven out of the 831 publications (4.5%) on DB-ACLR were classic articles with more than 100 citations. The highest number of citations (*n* = 352) was for the prospective cohort study from Hokkaido University School of Medicine, Sapporo, Japan (PMID: 16517306) published in Arthroscopy: The Journal of Arthroscopic and Related Surgery. The second highest number of citations (*n* = 289) were of the randomized controlled trial from Tokyo Medical and Dental University, Tokyo, Japan (PMID: 17560476) published in Arthroscopy: The Journal of Arthroscopic and Related Surgery. The third highest number of citations (*n* = 279) was from a randomized controlled trial (RCT) from Hyogo College of Medicine, Nishinomiya, Japan (PMID: 17091015) published in Clinical Orthopedics and Related Research. The bibliometric details of the top 10 publications on DB-ACLR are presented in Table 3. The top three articles that received the maximum number of citations (ranging from 279 to 352 citations) were written by Japanese authors.

**Table 3:** The 10 most popular articles on double-bundle ACL reconstruction (DB-ACLR)

Number	Authors, Journal, year	PMID	Country	Institution	Citation count
1	Yansuda et al., Arthroscopy, 2006	16517306	Japan	Hokkaido University School of Medicine, Sapporo	352
2	Muneta et al., Arthroscopy, 2007	17560476	Japan	Tokyo Medical and Dental University, Tokyo	289
3	Yagi et al., Clin Orthop Relat Res, 2007	17091015	Japan	Hyogo College of Medicine, Nishinomiya	279
4	Yamamoto et al., Am J Sports Med, 2004	15572308	USA	University of Pittsburgh, Pittsburgh, Pennsylvania	247
5	Zantop et al., Am J Sports Med, 2008	17932407	Germany	Wilhelms University Muenster, Muenster	243
6	Ahldén et al., Am J Sports Med, 2012	22962296	Sweden	Sahlgrenska University Hospital/Mölndal	238
7	Forsythe et al., J Bone Joint Surg Am, 2010	20516317	USA	University of Pittsburgh, Pittsburgh, PA	233
8	Siebold et al., Arthroscopy, 2008	18237696	Germany	ARCUS Sportsclinic, Pforzheim	229
9	Järvelä, Knee Surg Sports Traumatol Arthrosc, 2007	17216271	Finland	Hatanpää Hospital, Tampere	216
10	Hussein et al., Am J Sports Med, 2012	22085729	Slovenia	Artros Center for Orthopedic Surgery and Sports Medicine, Ljubljana	209

### Relative Citation Ratio (RCR)

The value of the relative citation ratio (RCR) was available for 8574 out of 9699 articles on SB-ACLR. The 8574 articles on SB-ACLR were influential, with a mean RCR of 1.882 ± 2.651. The article with the highest RCR of 48.96 was the most cited article on SB-ACLR (PMID: 17761605).

The value of RCR was available for 774 out of 831 articles on DB-ACLR. The 774 articles on DB-ACLR were influential, with a mean RCR of 1.792 ± 2.044. The article with the highest RCR of 14.90 was also the most cited (PMID: 16517306). This difference in mean RCR between SB-ACLR and DB-ACLR was not significant (*p* = 0.25; 95% CI − 0.07 to 0.25).

### Other Metrics

The median citations per year for SB-ACLR (median = 1.62; IQR: 0.5, 3.6) was lower than that of DB-ACLR (median = 1.80; IQR: 0.75, 3.57) and the difference was significant (*p* = 0.03).

The expected citations per year details were available for 8494 publications on SB-ACLR, and the expected citations per year details were available for 764 publications on DB-ACLR. The median expected citations per year for SB-ACLR and DB-ACLR were 1.61 (IQR: 1.45, 1.75) and 1.60 (IQR: 1.50, 1.70), respectively, and the difference was not significant (*p* = 0.36).

The field citation rate details were available for 8948 publications on SB-ACLR and 795 publications on DB-ACLR. The median field citation rate for SB-ACLR (median = 2.66; IQR: 2.28, 3.02) was higher compared to that of DB-ACLR (median = 2.54; IQR: 2.31, 2.82), respectively, and the difference was significant (*p* < 0.0001).

The NIH percentile was available for 8466 SB-ACLR publications and all 831 DB-ACLR publications. The median NIH percentile for SB-ACLR (median = 53.55; IQR: 22.0, 79.2) was higher compared to that of DB-ACLR (median = 51.8; IQR: 18.7, 77.4), respectively, and the difference was significant (*p* = 0.04).

### Data Mining

**Fig. 2:**
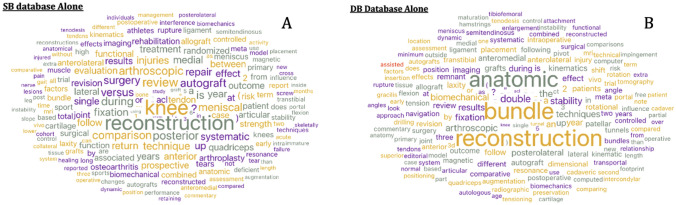
Word clouds from titles for **A**. Single bundle ACLR alone **B**. Double bundle ACLR alone. Output using Orange Software

**Fig. 3:**
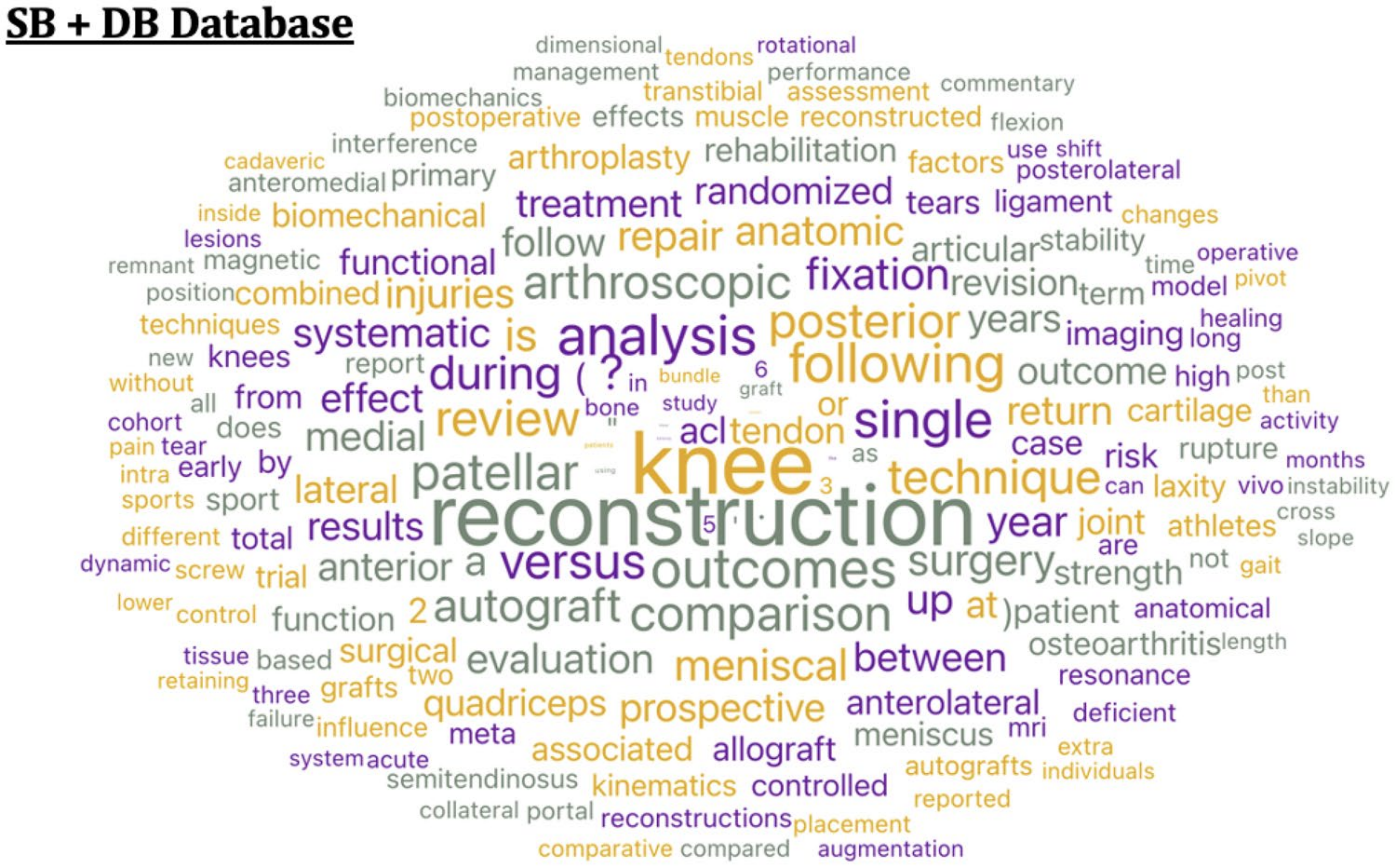
Word cloud from the titles of the combined database of SB and DB. Output using Orange Software

Figure 2A shows data mining for the database with SB articles alone. The occurrence of the words is given on the right side of the figure. On the left side, the word cloud depicts occurrences with increased font size indicating higher occurrences of the words. Terms toward the center are more commonly found in articles related to SB ACLR. Reconstruction, systematic, autograft, and meniscal are some of the prominent words in this search. In Fig. 2B, which depicts the word cloud and occurrence numbers for DB ACLRs, the words anatomic, bundle, stability, and biomechanical were seen more prominently. Figure 3 shows the word cloud output for the combined database of SB and DB. The prominent words here include reconstruction, outcomes, comparison, and versus Common words that build sentences were removed from the titles of all the databases.

## Discussion

The main important findings of the current study reveal that most of the last twenty years of scientific literature about ACL focused mainly on the single-bundle technique instead of the DB technique, with a ratio of almost 5–1 in the annual comparison of published articles. Currently, the academic output of SB ACLR remains at a high level, with about 900 articles published annually, while the same cannot be said for articles about DB ACLR, which do not even reach 100 publications per year.

The results of our study confirm our hypothesis and this is due to several different factors.

In the past, SB surgery has been a common surgical option to reconstruct torn ACLs. However, after ACL repair or other similar surgical treatments, degenerative alterations or arthrofibrosis continue to be a significant problem [25, 32]. After SB-ACLR, ineffective tibial rotation control has been linked to arthrofibrosis, a degenerative joint disease that results in osteoarthritis. Furthermore, according to recent biomechanical investigations, SB-ACLR is unable to restore healthy anterior translation or rotatory laxity [33]. DB-ACLR procedures are being promoted to more accurately mimic the native anatomy of the ACL and potentially increase the stability of the knee joint to further enhance the current SB-ACLR techniques and provide a better understanding of the ACL anatomy [34]. Although the reconstruction of both ACL bundles appears to have a theoretical advantage, there is still disagreement over whether DB-ACLR is better than the more common SB-ACLR [35]. The biomechanics of the ACL have been studied in numerous experimental settings throughout the last 10 years. However, questions about the methods chosen to restore normal knee biomechanics still exist [34]. One of the key disputes in ACLR relates to the importance of DB reconstruction in biomechanical outcomes compared with SB reconstruction, among other surgical issues such as graft types, fixation techniques, and bundle count [34].

One of the main factors that led to the greater development of the single-beam technique is certainly the distinctly different learning curves of the two techniques; Luthringer et al. conducted an assessment of the accuracy and precision pertaining to the placement of femoral and tibial tunnels in the context of anterior cruciate ligament reconstruction with the use of a single-bundle technique. This evaluation specifically focused on the utilization of an independent anteromedial portal (AMP). The study also revealed greater precision in the positioning of tunnels, as indicated by a decrease in standard deviations seen over the course of each year. A statistically significant improvement in the location of the femoral tunnel was observed when comparing the first and second cohorts, each consisting of 32 cases. There was no substantial alteration found in the tibial tunnel angle throughout the duration of the study [36].

In a study conducted in 2010, Snow et al. investigated the ability of a skilled ACL surgeon to transition from the SB approach to the DB procedure with a reasonable level of precision. No complications were observed. The duration of surgery for the first patient was recorded at 125 min, while the tenth patient's surgery lasted for 65 min [37].

In 2020, Oh et al. conducted a meta-analysis to compare the biomechanical results of SB and DB ACLR. Their findings indicate that both procedures are related with the restoration of normal knee kinematics. The efficacy of DB-ACLR surpasses that of SB-ACLR in the context of anteroposterior stability restoration. Nevertheless, the optimal strategy for achieving more enhancement in internal rotation laxity during a simulated pivot shift at a given angle has yet to be definitively determined [20].

In their study, Chen et al. conducted an analysis of the mid- to long-term outcomes of SB and DB anterior cruciate ligament ACLR. The study aimed to evaluate knee stability, clinical function, graft failure rate, and osteoarthritis (OA) changes. The researchers concluded that the DB technique did not demonstrate superiority over the SB technique in autologous ACL reconstruction when considering knee stability, clinical function, graft failure rate, and OA changes during mid- to long-term follow-up [19].

Similarly, Mayr et al. conducted a comparative analysis between anatomic SB and DB ACLR techniques, assessing the corresponding clinical outcomes after a 5-year follow-up period. The researchers reached the conclusion that after a 5-year follow-up, there is no discernible advantage for either the DB or SB approach in ACLR when considering patient-related and objective outcome indicators [38].

In contrast, Anandan et al. found that DB-ACLR yields enhanced rotational stability and superior functional outcomes, specifically in terms of returning to the preinjury activity level, as compared to SB-ACLR. The utilization of hamstring tendon autografts in DB-ACLR yields superior functional outcomes throughout the 10-year postoperative evaluation [39].

Another fact that favors the higher number of publications and therefore operations performed with the single-bundle technique concerns postoperative pain: recently, Chuaychoosakoon et al. evaluated differences in postoperative pain between SB and DB-ACLR with a hamstring graft in a retrospective study. The SB group exhibited lower average postoperative pain levels at all measured time periods. The results of the linear mixed-effect regression analysis indicated that, after accounting for confounding variables, the SB group exhibited lower levels of postoperative discomfort compared to the DB group. However, there was no statistically significant disparity observed between the two groups in terms of the number of bundle ACLRs with respect to morphine consumption [40].

However, a recent statistical study strongly criticized studies comparing the single- and double-beam techniques in ACLR. Ehlers et al. examined the statistical stability of studies comparing primary single-bundle to double-bundle ACLR utilizing autograft and independent tunnel drilling. The authors described two new metrics: the Fragility Index (FI) and Fragility Quotient (FQ). The FI for each binary outcome is defined as the change in the number of events that are required to change the status of statistical significance. The FI is an indicator of the statistical strength of the data. The FQ is defined as the percentage of change in the events that are required to change the status of statistical significance. Higher values of FI and FQ indicate statistical stability and robustness whereas lower values of FI and FQ suggest weakness and lack of statistical robustness (statistical fragility or statistical weakness). Out of the total number of 1794 studies that underwent screening, a subset of 15 comparative studies were selected for further research. Among these, 13 studies were identified as randomized controlled trials (RCTs). In general, the average fragility index (FI) and fragility quotient (FQ) were calculated to be 3.14 and 0.050, respectively. In 72.9% of cases, the number of patients lost to follow-up exceeded the FI. The statistical stability of studies comparing SB-ACLR with DB-ACLR may be less robust than previously assumed. Comparative studies and RCTs are susceptible to statistical fragility, as even a small number of event reversals can significantly impact the observed significance. The statistical significance of a given finding can be affected by the reversal of fewer than four outcome events in a therapy group, which is often lower than the number of patients lost to follow-up [41].

Currently, a variety of methodologies are accessible for implementation in routine clinical practise. The present study's findings provide valuable insights into the historical development of ACLR, specifically focusing on the two most commonly employed techniques: single and double-bundle. This study offers a comprehensive overview of the past and current interest in the diverse strategies that have been developed over time. Moreover, it highlights the key strategies that clinicians should consider in this field. Furthermore, our bibliometric research not only yielded valuable insights into the historical progression of ACL surgery, elucidating the patterns of the most influential techniques employed in the past, but also discerned the most auspicious and burgeoning treatments. The increasing scholarly endeavors, along with the emergence of novel technology, have the potential to facilitate significant advancements in the pursuit of improved treatments for the restoration of the ACL.

This bibliometric study presents some limitations; e.g., we only included articles that were published in the PubMed database, and hence, some of the journals (publishing on this topic) that were not included in PubMed were excluded. The search strategy may have included some articles with double-bundle MCL and PCL reconstructions along with SB-ACLR. However, the number of these articles appears to be negligible and does not affect the overall trends.

## Conclusion

In this bibliometric study, we found a downward trend of publications on double-bundle ACLR, after reaching a peak in 2012, but the publications related to single-bundle ACLR have continued to increase. Despite the theoretical superiority of DB-ACLR in providing more rotational stability than SB-ACLR, its popularity has peaked and is on decreasing trend now. The number of publications on single-bundle ACLR was significantly higher than that on double-bundle ACLR over the last 20 years.

## Data Availability

The raw data are available with us if needed.

## Code Availability

The datasets generated during and/or analyzed during the current study are available from the corresponding author on reasonable request.
